# Pattern Mining-Based Pig Behavior Analysis for Health and Welfare Monitoring

**DOI:** 10.3390/s24072185

**Published:** 2024-03-28

**Authors:** Hassan Seif Mluba, Othmane Atif, Jonguk Lee, Daihee Park, Yongwha Chung

**Affiliations:** 1 Department of Computer and Information Science, Korea University, Sejong City 30019, Republic of Korea; 2017011010@korea.ac.kr (H.S.M.); osuman@korea.ac.kr (O.A.); 2 Department of Computer Convergence Software, Sejong Campus, Korea University, Sejong City 30019, Republic of Korea; ychungy@korea.ac.kr

**Keywords:** pig behavior recognition, pig behavior analysis, sequential pattern mining, association rule mining, data visualization, pig health and welfare

## Abstract

The increasing popularity of pigs has prompted farmers to increase pig production to meet the growing demand. However, while the number of pigs is increasing, that of farm workers has been declining, making it challenging to perform various farm tasks, the most important among them being managing the pigs’ health and welfare. This study proposes a pattern mining-based pig behavior analysis system to provide visualized information and behavioral patterns, assisting farmers in effectively monitoring and assessing pigs’ health and welfare. The system consists of four modules: (1) data acquisition module for collecting pigs video; (2) detection and tracking module for localizing and uniquely identifying pigs, using tracking information to crop pig images; (3) pig behavior recognition module for recognizing pig behaviors from sequences of cropped images; and (4) pig behavior analysis module for providing visualized information and behavioral patterns to effectively help farmers understand and manage pigs. In the second module, we utilize ByteTrack, which comprises YOLOx as the detector and the BYTE algorithm as the tracker, while MnasNet and LSTM serve as appearance features and temporal information extractors in the third module. The experimental results show that the system achieved a multi-object tracking accuracy of 0.971 for tracking and an F1 score of 0.931 for behavior recognition, while also highlighting the effectiveness of visualization and pattern mining in helping farmers comprehend and manage pigs’ health and welfare.

## 1. Introduction

In South Korea, pork is the most popular meat owing to various factors including its wide availability and affordability as a source of protein [1,2]. This popularity has resulted in a notable rise in pork consumption over the past few years, which grew from 1423.51 thousand tons in 2006 to 2081.61 thousand tons in 2021, corresponding to a total increase of 46% in pork consumption [3]. To fulfill the continuously growing demand, pig farmers have been expanding their farming operations [4], which has contributed to the increase in the number of pigs raised. For example, the number of pigs raised in farms in South Korea grew from 9.38 million in 2006 to 11.22 million in 2021, representing a total increase of 20% [5]. However, the number of pigs raised in farms is increasing while the number of farm workers is decreasing due to factors including aging of the farm population and the movement of young individuals from rural villages to urban areas, seeking more financially rewarding job opportunities, which make it difficult to recruit new workers [6]. The proportion of agriculture workers to the total population fell from 8% in 2006 to 5% in 2021 [7], contributing to the shortage of the agricultural workforce [8]. Due to this shortage, the available farm workers are assigned greater workloads to finish within limited time frames, rendering effective performance of their tasks challenging. These tasks include the management of health and welfare issues, reproduction and breeding, nutrition and feeding, and waste management [9,10,11]. Among these, the most critical issue is the management of pigs’ health and welfare [12,13] as this issue can lead to high mortality rates, low growth rates, weight loss, injuries, and an increase in veterinary costs [10,14,15,16]. This ultimately results in low productivity and imposes financial burdens on pig farmers [12]. Hence, to maintain productivity and minimize financial losses, it is essential for pig farmers to manage their pigs’ health and welfare.

Continuous monitoring of pigs’ behaviors is crucial for farmers to better manage their pigs’ health and welfare [17], because it can assist them in identifying subtle changes in behaviors that occur before or alongside subclinical and clinical signs of disease or injury. Ultimately, such monitoring can help farmers understand their pigs’ health and welfare issues [18,19]. Traditionally, the monitoring of pigs’ behaviors in farms is conducted manually. However, with this approach, pig farmers need to continuously observe pigs and keep track of their displayed behaviors, which is time-consuming [17], error-prone [20], and impractical [21], considering that the number of farm workers is limited. To address these issues, automatic monitoring of pigs’ behaviors is more appropriate as it enables a more reliable and accurate continuous observation without much need for intervention from farm workers. Therefore, in this study, we propose a method to automatically monitor and identify individual pigs, recognize their behaviors, and analyze them to provide pig farmers with important information that can help them effectively understand and manage pigs’ health and welfare.

Recently, several studies [22,23,24,25,26,27,28,29,30,31] have reported the use of a technology-driven approach to automatically and continuously monitor and manage animals’ health, welfare, breeding, and production and reproduction using various sensors in what is known as “Precision Livestock Farming” (PLF) [32,33,34,35,36]. Utilizing video sensors, PLF methods have been proven to be efficient, noninvasive, and stress-free, which allows continuous, real-time, and automated monitoring of animals to assist farmers in managing their livestock and improving their productivity. Considering these benefits, some studies [37,38,39,40,41,42,43,44] have leveraged PLF methods with video data to recognize pigs’ behaviors relevant to their research. Table 1 summarizes some of the recent studies related to pig behavior recognition. Some researchers, such as Nasirahmadi et al. [37], used traditional machine learning techniques because they need less data to train the model and are less computationally demanding. Meanwhile, others [38,39,40,41,42,43,44] used deep learning techniques since they can automatically extract more relevant features (instead of relying on handcrafted features), which improves performance. Before performing behavior recognition, some of the aforementioned studies used predefined bounding boxes and cropped images to localize pigs for validating their approach in recognizing individual postures in commercial farms [37] and providing a low-cost solution to monitor the aggressive behavior [40] as well as feeding and drinking behaviors [44] of individual pigs. By contrast, other studies used detection techniques to automate the process of localizing individual pigs for monitoring their drinking, urination, and mounting behaviors in real time [38], monitoring and assessing their postures in response to various heat conditions [41], and improving the performance of individual posture recognition [42,43]. In addition to using a detection method to automatically localize pigs, Alameer et al. [39] used tracking to identify individually detected pigs using unique identifiers (IDs) for differentiating the pigs and keeping a record of the behaviors displayed by each pig along with their IDs.

Even though these studies have demonstrated good performance, most of them are mainly focused on providing recognized behaviors to help farmers manage their pigs. In practice, however, if farmers are solely provided with the recognized pigs’ behaviors, they need to read through the behavior logs manually to gain insight on the pigs’ health and welfare. This requires a significant effort and consumes a considerable amount of time that can result in missing out on important information that would assist them in managing their pigs [45]. Instead of making farmers use this approach, it is more efficient and accurate to analyze the recognized behaviors and provide farmers with the extracted information that can deliver valuable behavioral patterns related to the pigs’ health and welfare. This will help farmers to better understand the state of their pigs, enabling effective management while also reducing labor costs and time [46], ultimately leading to pigs’ better health and welfare and improved farm productivity [47].

Various methods can be used to analyze pigs’ behaviors and extract useful information and patterns from them. For example, Alameer et al. [39] used descriptive statistical analysis to analyze pigs’ behaviors and provide pig farmers with graphical representations that enable them to visualize the duration that pigs spent in different postures and drinking behaviors, as well as the pen positions where those behaviors occurred. Even though this method makes it easier for pig farmers to view the displayed behaviors and assist them in performing an initial analysis, their main purpose is summarizing data to produce a visual representation, which still requires farmers to manually search for patterns. To provide farmers with a more detailed behavioral analysis that can assist them in effectively understanding and monitoring their pigs’ health and welfare, it is crucial to apply techniques that can automatically process pigs’ behavioral data and discover hidden, understandable, and valuable patterns from them. Pattern mining techniques are a suitable solution as they not only offer the aforementioned benefits but can also provide relationships and unexpected or previously unknown insights from large datasets that can be difficult to extract using manual observations and other analytical techniques [48,49]. To exploit these advantages, some researchers have utilized pattern mining methods to analyze animal behaviors. For example, Fontes et al. [50] analyzed jaguar movement data using association rule mining to uncover their social interactions and relationships, which are vital for understanding their ecology. Furthermore, Branco et al. [51] used the generalized sequential pattern (GSP) algorithm to identify and characterize the sequential behavior patterns of broiler chickens under different heat conditions as an initial step in developing a smart environment-control system. Likewise, applying pattern mining techniques to pigs’ behaviors can help uncover valuable information, such as the time-ordered sequences and interdependence among behaviors, which can serve pig farmers in managing and improving their pigs’ health and welfare. In fact, the pigs’ behavioral sequential patterns can provide insights on the type and duration of behaviors that lead to aggression, revealing their causes [52] and severity [53], helping farmers understand and address them appropriately. For example, if there is a sequence of aggressive and feeding behaviors in the mined behavioral sequential patterns, this may indicate that poor feeding conditions and a lack of proper nutrients contribute to the aggression among pigs [54,55]. This information will facilitate the development of evidence-based interventions to improve the feeding environment and the type of food to reduce the pigs’ aggressive behavior [54,55]. Furthermore, behavioral sequential patterns can offer insights about the necessity of providing pigpens with access to toys and devices (known as environmental enrichment [56]) to keep the pigs entertained, which will reduce stress and aggressive behavior. For instance, if the mined behavioral sequential patterns include activeness behavior followed by bar-biting in sequence, this may suggest poor environmental conditions or inadequate enrichment materials in the pigpen [57]. This can help pig farmers understand and address the issues to alleviate pigs’ stress and frustration, as well as aggressive behavior [58,59,60], which will enhance their health and welfare. Moreover, the association between pigs’ behaviors can help identify patterns and relationships, contributing to improved decision-making in domains like nutrition, housing, and health and welfare management. For instance, if “moving” behavior is associated and positively correlated with “resting” behavior, it implies that active pigs have adequate time to rest, which reveals good welfare among the pigs [61]. This will eventually allow pig farmers to maintain their living conditions and improve their productivity. Therefore, it is essential to use pattern mining techniques to analyze pigs’ recognized behaviors as they can extract patterns that will assist pig farmers in making well-informed management decisions regarding pigs’ health and welfare.

Lastly, only a few studies have utilized both detection and tracking techniques to localize and identify individual pigs before behavior recognition [39]. However, pig detection and tracking are an essential initial step for recognizing individual pigs’ behaviors in a pigpen with multiple pigs. In addition, pig detection and tracking information can add value to the understanding of pigs’ health and welfare. For instance, analyzing pigs’ trajectory information using descriptive statistical techniques can provide visualized locomotion patterns based on the distance they have covered, which can help to evaluate their health status [62]. Thus, to address some of the limitations of previous research, in this study, we propose a system that acquires video data of pigs, detects and tracks them, recognizes their behavior, and analyzes the tracking and recognized behavioral data. Descriptive statistical analysis is first used to provide a general visualization for a quick initial exploration, and then pattern mining techniques are used to perform deeper analysis and automatically extract patterns. This provides pig farmers with meaningful information that helps them to manage their pigs’ health and welfare. The system comprises the following features:

(1) Acquiring pig video data.
(2) Automatically detecting and tracking individual pigs.
(3) Recognizing the behavior of individual pigs.
(4) Analyzing tracked and recognized behavioral data, which, in turn, provide pig farmers with extracted information that will assist them in making well-informed management decisions regarding their pigs’ health and welfare.

Therefore, in this study, we propose a pattern mining-based system to analyze pigs’ behaviors to assist farmers in effectively monitoring the health and welfare of their pigs. In our proposed system, we first employ the ByteTrack [63] method, which is composed of YOLOx as the detector and the BYTE algorithm as the tracker, for the detection and tracking of pigs, respectively. This method demonstrates strong performance and effectively addresses challenges such as occlusion, motion blur, size changes, and long-range association between detected and tracked objects. Then, we extract the appearance features for each tracked pig using the Mobile neural architecture search Network (MnasNet) [64], which is a convolutional neural network (CNN) that performs better in terms of accuracy and inference latency. Subsequently, we utilize long short-term memory (LSTM) to extract temporal information and recognize the pigs’ behaviors. Following this, the tracking information and recognized behaviors are captured and stored as log data. Subsequently, we apply descriptive statistical analysis and pattern mining techniques to extract visualized information and behavioral patterns that provide insights on the pigs’ health and welfare.

The rest of this paper is organized as follows. In Section 2, we describe in detail the method proposed in this study. Section 3 presents the experimental results and their significance. Section 4 discusses the implication of the findings in a broader context. Lastly, in Section 5, we conclude our study and present recommendations for future work.

## 2. Proposed Method

**Figure 1:**
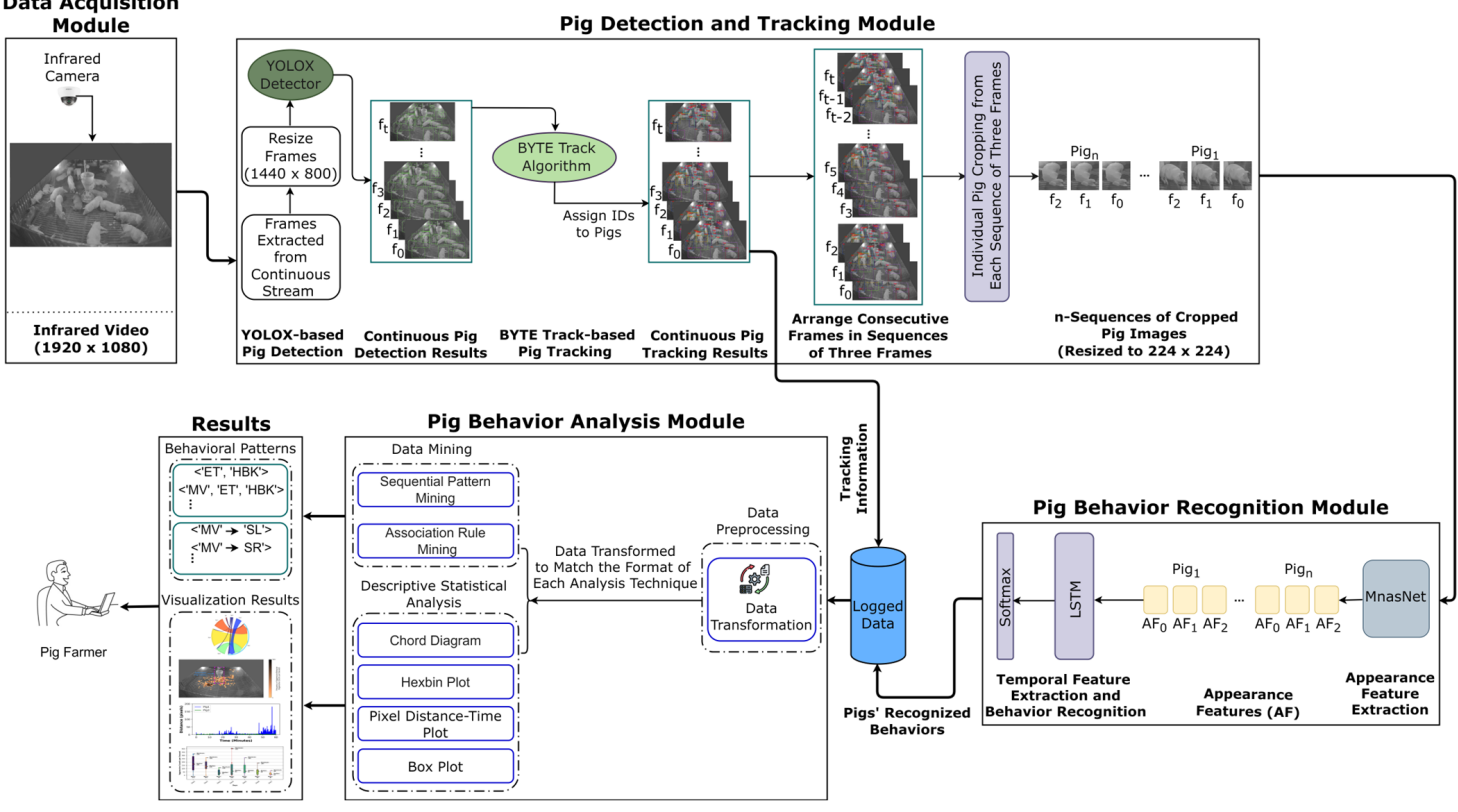
Overall structure of the pattern mining-based pig behavior analysis system.

The proposed architecture for the pattern mining-based pig behavior analysis system is depicted in Figure 1. The system consists of four main modules: (1) data acquisition module, (2) pig detection and tracking module, (3) pig behavior recognition module, and (4) pig behavior analysis module.

### 2.1. Data Acquisition Module

The data acquisition module receives a continuous stream of video data from an infrared camera installed at the top of the pigpen, which continuously monitors multiple pigs inside it. An infrared camera was selected due to its capability of capturing video even in low-light conditions, rendering it suitable for 24 h continuous monitoring of pigs in a pigpen [26]. Subsequently, the received data were forwarded to the next module to detect and track the pigs.

### 2.2. Pig Detection and Tracking Module

The second module in our proposed system is the pig detection and tracking module, which focuses on localizing and uniquely identifying multiple pigs individually in the pigpen. This step is essential as it forms the basis for extracting information about the pigs’ location and unique IDs, which play a crucial role in the subsequent behavior recognition and analysis stages. Given the importance of this task, it is necessary to use a method that can effectively handle Multiple Object Tracking (MOT) and provide good results with multiple pigs. In general, there are two types of MOT methods, namely Detection-Free-Tracking (DFT) and Detection-Based-Tracking (DBT) methods [65]. DFT methods focus on associating and tracking a fixed number of objects across consecutive frames without relying on explicit detection results. By contrast, DBT methods have the ability to handle a varying number of objects by detecting them in each frame and performing data association across video frames to provide accurate and precise tracking results [65]. This feature makes DBT methods suitable for tracking multiple pigs in a pigpen. Several DBT methods exist [66,67,68,69,70] and have been applied in animal-related studies [21,71], achieving good results and accurately tracking multiple animals simultaneously. However, a notable limitation of these methods is their failure to consider objects with low detection scores, which can raise false negative values and reduce their overall performance. In our case, this will lead to errors in pig detection and tracking, ultimately affecting behavior recognition and analysis results in the later stages. Therefore, in our proposed system, it is also important to consider pigs with low detection scores while tracking them to ensure good tracking performance. After reviewing recent studies on MOT, we selected the ByteTrack method proposed by Zhang et al. [63], which effectively addresses the issue caused by objects with low detection scores. First, this method uses a high-performance anchor-free detector, YOLOX [72], for detecting objects, and then employs the BYTE track algorithm to associate detected objects with their tracking IDs by considering every detection box, including those with a score lower than the selected threshold value. In this manner, the ByteTrack method enhances MOT performance and addresses issues such as occlusion, motion blur, size-changing, and long-range association between the detected and tracked objects, ultimately achieving good results compared with other tracking algorithms [73,74,75]. For these reasons, in this module, we used the ByteTrack method, which is composed of YOLOX and the BYTE algorithm, for the detection and tracking of pigs from the received stream of videos.

To apply the ByteTrack method for detection and tracking of multiple pigs in the pigpen, first, single-channel grayscale frames were extracted from the received stream of pig videos and transformed into three-channel red–green–blue (RGB) frames by replicating the single-channel data into two additional channels. This ensures that the extracted frames are in the form of three-channel RGB frames, which adhere to the input requirements of YOLOX-x used in this module and the MnasNet model used in the next module [63,64]. Next, the newly obtained RGB frames were resized to a resolution of 1440×800 pixels to align with the input size of the YOLOX-x model before being forwarded for pig detection. Following this, the detection score for each detected pig was compared with the detection threshold value. If the score was greater than the threshold value, the detected pig was grouped as a high-confidence detection; otherwise, the detected pig was grouped as a low-confidence detection. At this stage, the BYTE track algorithm first assigned a tracking ID to the pigs with high scores and performed an association across the video frames. If the tracking ID remained unmatched with some detection boxes across the frames, then the algorithm associated them with low-score pigs to recover their tracking IDs. Subsequently, the individual frames (each containing pigs with bounding boxes and unique IDs) were outputted and arranged in sequences of three frames to align with the input size required by the behavior recognition model used later. Meanwhile, the pigs’ bounding boxes and IDs were stored as tracking information in the logged data. Furthermore, the bounding boxes were used to determine the regions of interest (ROIs) for each pig in the pigpen, which were then used to crop their image, enabling behavior recognition of specific individual pigs. This resulted in a total of n sequences of cropped images for each set of three frames, where n represents the number of pigs in the pigpen. Lastly, the cropped images were resized to a resolution of 224×224 pixels to match the input size of the next model before being forwarded for behavior recognition of every pig.

### 2.3. Pig Behavior Recognition Module

This module receives sequences of three cropped pig images as inputs from the detection and tracking module to recognize each pig’s behavior. In this context, the term “pig behavior” includes both normal behaviors (e.g., sleeping and moving) and aggressive behaviors (e.g., tail-biting and ear-biting), which are further elaborated in detail in Section 3.1.

Behavior recognition is a field of research that has garnered much interest in past few years due to its potential for diverse applications, including surveillance, anomaly detection, and monitoring of animal health and welfare. To ensure good analysis of recognized behaviors, the selection of a suitable method that provides precise results in pig behavior recognition is of paramount importance, as subsequent steps depend on the accuracy of this phase. Accordingly, we explored recent approaches for behavior recognition in video data and found that deep learning methods, particularly the combination of CNN and LSTM (CNN–LSTM), have gained popularity and have been applied in various studies [29,37,76,77,78,79]. The CNN–LSTM model allows automatic extraction of more relevant features by effectively extracting appearance information from images using CNN, which are then forwarded to the LSTM model to capture temporal dependencies in sequential data. This enables the model to learn complex representations of the behaviors to be recognized, which improves the performance of the model [40,80]. Thus, in this module, we used a CNN–LSTM model for pig behavior recognition.

Various CNN architectures can be used in the CNN–LSTM model for appearance features extraction from images. For example, VGG16 was used as an appearance feature extractor in VGG16–BiLSTM to recognize cows’ basic behaviors in order to understand their physiological health status. Similarly, Chen et al. [81] employed ResNet50 as the appearance feature extractor in ResNet50–LSTM to classify the drinking and drinker-playing behaviors of pigs to gain insight into whether the pigs had adequate water consumption. These CNN models have demonstrated their effectiveness in extracting representative features for CNN–LSTM models, enabling accurate behavior recognition. However, to ensure improved results in pig behavior recognition, we explored and considered various recently developed and improved CNN models that can effectively extract representative features for behavior recognition in CNN–LSTM models. Among these models is the MnasNet, which uses a neural architecture search (NAS) technique and a depth multiplier (DM) value to automatically reduce the number of parameters in order to produce models with optimal network architectures that are lightweight and efficient [64]. The MnasNet model has been proven to be effective [82] as it is small in size, lightweight, and performs better in terms of accuracy and inference latency, which, in our case, can ensure precise recognition of different pigs’ behaviors while enabling fast inference and timely response. For these reasons, in our CNN–LSTM model, MnasNet was used to extract appearance features from the received sequences of cropped pig images. To use the MnasNet model, the DM value was first set at 0.5 to reduce the model size and make it lighter by reducing the number of filters in each layer by half. This model is referred to as the MnasNet0.5 model. Subsequently, the classifier layer was removed to use the model as an appearance feature extractor on the received sequences of three cropped pig images from the previous module. This image sequence length was selected because it yielded better results for pig behavior recognition compared with other image sequence lengths, as demonstrated in the ablation study (Section 3.3.2). Next, the extracted feature vectors were forwarded to the LSTM as inputs for temporal feature extraction, followed by pig behavior recognition through a softmax layer classifier. Lastly, the pigs’ recognized behaviors were saved as log data to be used for analysis in the next module.

### 2.4. Pig Behavior Analysis Module

In this module, descriptive statistical analysis and data mining techniques were applied to extract visualized information and behavioral patterns, providing a deeper understanding of pigs’ behaviors, thereby enabling informed decisions that can enhance their well-being as well as overall management practices. To apply these techniques, the tracking information and recognized behaviors captured and stored as log data were first loaded and preprocessed by transforming them according to the specific format required for each analysis technique. Subsequently, descriptive statistical analysis and data mining techniques were applied to the preprocessed data to generate visualized information and extract behavioral patterns, which were then sent to the farmer to better understand and gain insights into the pigs’ health and welfare. Chord diagram, hexbin plot, pixel distance–time plot, and box plot were employed for descriptive statistical analysis while the sequential pattern mining and association rule mining techniques were used for data mining. The process for each analysis technique is presented in more detail in the following subsections.

#### 2.4.1. Analysis of Aggressive Pigs’ Relationships

Pig aggression on farms typically arises from a minority of individuals exhibiting aggressive behaviors [83], and identifying these individuals manually is a challenging and time-consuming task. If pig aggression is not effectively addressed, it can lead to unfortunate outcomes such as the death of victim pigs due to fatal attacks. Therefore, it is imperative to identify both aggressors and victims within the pigpen, along with their associated aggressive relationships, in order to implement appropriate measures and ultimately prevent further instances of aggression [84,85,86]. To assist pig farmers with this issue, our system uses the tracking information and recognized behaviors to identify aggressor and victim pigs, delineate their aggressive relationships, and quantify the degree of aggression (such as which pigs are most targeted and which ones initiate attacks the most within the pigpen). Following this, the system presents this information in a visualized form using a chord diagram since it can display interactions among multiple elements, the extent of these interactions, and the initiators and receivers in a clear and intuitive manner. By employing this method, pig farmers can easily distinguish aggressors from victims, understand which animals are most frequently involved in attacks, and subsequently take action to resolve the problem.

#### 2.4.2. Analysis of Locations Where Aggressive Behaviors Occur

Identifying the key locations where aggressive behaviors take place is of great importance because it can help pig farmers understand potential underlying causes (e.g., competition for resources and social hierarchies) and enable them to take necessary actions to address these issues [87]. To achieve this, our system uses bounding boxes of aggressive pigs and presents them in a hexbin plot, since it effectively provides visualization of locations where aggressive behaviors occur by aggregating trajectory points and color-coding them based on frequency. Therefore, providing farmers with a hexbin plot can help them to effectively visualize the key locations where aggressive behaviors occur, enabling a better understanding of their potential underlying causes, and subsequently implementing the necessary measures to address them.

#### 2.4.3. Analysis of Pigs’ Movements

Understanding pigs’ movements can provide insights into their health and welfare. In fact, pigs that exhibit abnormal or restricted movement patterns may suggest underlying problems that require attention [11]. For instance, if pigs experience or show difficulties in moving or walking properly, this may indicate reduced or no activity, which might be a sign of lameness and sickness [88]. Moreover, a decrease in movement may imply health issues or environmental stressors that limit the pig’s mobility and exploration [59]. To assist farmers with this issue, our proposed system uses tracking information and employs a pixel distance–time plot, providing a clear visualization of the pigs’ movements, allowing for effective observation and identification of any signs that may reveal issues that need to be addressed.

#### 2.4.4. Analysis of Aggressive Behavior Variations

The variations or changes in the exhibited behaviors of pigs over time can be attributed to various factors, including health, environment, social interactions, and developmental stages [19,89,90,91]. Understanding these behavioral variations can assist pig farmers in improving animal welfare, optimizing management practices, and ensuring the long-term sustainability of pig farming. For instance, an increase in pigs’ aggressive behavior with respect to time may suggest several potential factors or issues including unstable social groups and inadequate nutrition [92]. By contrast, a decrease in pigs’ aggressive behavior can be interpreted as positive developments including adequate space and resources, establishment of social hierarchy, and environmental enrichment [16,53,87]. Therefore, our proposed system provides a box plot to delineate the trends in pigs’ aggressive behavior with respect to time. This plot allows farmers to effectively observe and comprehend variations in the pigs’ aggressive behavior over time, enabling them to implement necessary measures for handling pigs’ aggression.

#### 2.4.5. Pigs’ Behavioral Sequential Patterns

Pigs’ behavioral sequential patterns can help identify deviations or abnormalities that can signal potential problems or areas of concern to the pigpen. For instance, a sequence of “restlessness” behavior followed by “bar-biting” or engaging in stereotypic behaviors may indicate poor environmental conditions or inadequate enrichment in the pigpen [57]. In addition, if a pig vocalizes or squeals, followed by aggression toward its pen mates, this may imply social stress or hierarchical conflicts within the group in the pigpen [87]. Therefore, in this module, a sequential pattern mining technique was used to identify and extract sequential patterns, which can help in understanding and addressing potential issues that can contribute to the overall well-being, productivity, and health of pigs in the pigpen.

Various sequential pattern mining techniques exist in the literature [93,94,95,96,97,98,99] and their benefits have been demonstrated in several studies [51,100]. However, most of these techniques did not consider the constraints that specify the desired properties of the item attributes for generated patterns, leading to a large number of sequential patterns that may not provide relevant results to the user. To ensure that the farmers can effectively filter out the relevant and meaningful patterns, it is important to restrict the search of the sequential pattern mining algorithm to smaller subsets that satisfy problem-specific constraints. For instance, a farmer may seek to generate sequential patterns for pig behaviors exhibited in a sequence for a certain minimum amount of time to understand their impacts or severity, thereby helping them take appropriate action for managing their pigs. Accordingly, after reviewing recent studies on sequential pattern mining techniques, we selected the constraint-based sequential pattern mining algorithm proposed by Hosseininasab et al. [101]. This method effectively addresses the aforementioned issue by relying on a multi-valued decision diagram (MDD) representation of the database, accommodating multiple item attributes and constraints. Compared with other methods, this method is efficient and scalable because it can restrict the mining search to smaller subsets of patterns that satisfy problem-specific constraints, enabling pig farmers to extract desired patterns based on identified constraints. Thus, the constraint-based sequential pattern mining algorithm was applied in this study to extract sequential patterns from the recognized pigs’ behaviors.

To mine sequential patterns, first, the support and constraint threshold values were set on the constraint-based sequential pattern mining algorithm. Then, the algorithm was applied to the received pigs’ behavioral data to generate sequential patterns. These generated patterns are essential as they can help identify potential issues, thereby assisting pig farmers in addressing them and ultimately contributing to the well-being of the pigs in the pigpen.

#### 2.4.6. Pigs’ Behavioral Association Rule Mining

Association rule mining focuses on discovering patterns with interesting relationships and correlation among a large set of data items based on a defined threshold value [48,102]. In the context of pigs’ captured data, an association rule between exhibited behavior can be expressed in the form of X→Y, where X is the behavioral-antecedent and Y is the behavioral-consequent of the rules. Understanding pigs’ behavioral patterns can help in addressing issues by providing early intervention and appropriate management strategies and eventually improve their living conditions, and promote good health and welfare. For example, if pigs’ “being-attacked” behaviors are associated and correlated with their “sitting-resting” behaviors, it can reveal the consequences of aggression to the victim pigs that exhibit “resting” behavior due to the effects of the attack such as injuries, lesions, and pain [103]. In addition, when “moving” is associated with “eating” behavior, this may indicate good welfare among pigs as they need to actively search for food, access feeding areas, and engage in natural foraging behaviors [104]. To help farmers understand and deal with these issues, our system leverages the association rule mining approach, specifically the frequent pattern (FP) growth algorithm [105], to provide associative patterns that can assist farmers in identifying meaningful relationships among pigs’ behaviors, making it easier to explore and draw insights from them. Compared with other available methods, the FP growth algorithm is faster, more efficient, and scalable for association rule mining from both long and short frequent patterns, owing to its elimination of the need for the costly candidate set generation and testing approach, which is typically required for large and long patterns [106]. This makes the FP growth algorithm a suitable choice for analyzing pigs’ behaviors as it can be applied in a broad range of domains and guarantees the discovery of meaningful associative patterns from large datasets in a short amount of time.

To extract associative behavioral patterns, the support, confidence, and lift threshold values were first set on the FP growth algorithm [105]. Then, the algorithm was applied to the received pigs’ recognized behaviors to generate associative patterns. These generated patterns can assist farmers in identifying health and welfare issues, enabling prompt intervention and implementation of appropriate management strategies.

## 3. Experimental Results

### 3.1. Data Collection and Datasets

The video datasets for our study were collected from a pig farm in Hadong-gun, Gyeongsangnam-do, South Korea, using an infrared dome camera (QND-6012R, Hanwha Techwin Co., Changwon, Republic of Korea). The infrared dome camera was positioned at the top of the pigpen to record the video at ten frames per second with a resolution of 1920×1080 pixels. During the recording process, each video was recorded for a maximum of one hour, enabling the collection of a large number of samples showcasing a diverse range of pig behaviors. Subsequently, the videos containing pigs exhibiting selected aggressive and potentially harmful behaviors, as well as their common daily life behaviors, which can help farmers to enhance the understanding and management of their pigs’ health and welfare, were chosen and annotated. The Video Tracking and Behavior Annotation Tool (ViTBAT) was used to annotate the videos since it allows the annotation of both bounding boxes and IDs for object detection and tracking, as well as behaviors for behavior recognition experiments [107]. In this step, each pig in a frame was annotated with a unique ID and bounding box to create a training set for the detection and tracking model and further annotated with their associated exhibited behavior for the behavior recognition experiment.

To train the detection and tracking model, 21,348 frames were annotated. Out of these, 13,779 frames were allocated for training and validation sets in an 8:2 ratio. The remaining 7569 frames derived from five video sequences that showcased various conditions, including instances of occlusion as well as active and inactive pig movements, were used to test the generalization capability of the model. For the behavior recognition experiment, a total of 17,213 frames were annotated, resulting in 367,002 labeled behavior instances, as each frame contained multiple pigs, and each pig was considered as a separate instance. To determine the appropriate image sequence length used as the input for the MnasNet0.5–LSTM model for behavior recognition, an ablation study was conducted using various image sequence lengths on our pig behavior recognition dataset. The findings, presented in Section 3.3.2, show that an image sequence length of three images yielded better results compared with other tested image sequence lengths. Accordingly, sequences of three images were selected as the input for the MnasNet0.5–LSTM model, resulting in 122,334 instance sequences. From these instance sequences, 70, 15, and 15% were used for training, validation, and testing, respectively. Table 2 shows the description of pigs’ behaviors and their corresponding dataset counts used in the behavior recognition experiment as arranged in a sequence of three images.

### 3.2. Experimental Environment and Setup

A desktop computer running on Windows 10 operating system, with a 4.0 GHz Intel Core i7 central processing unit (CPU), 32 GB random access memory (RAM), and an Nvidia GeForce RTX 3080Ti graphics card (ZOTAC Technology Limited, Hong Kong, China), was used for all our experiments. All models were implemented and trained in Anaconda environment using Python 3.8 programming language and Pytorch 1.7 open-source machine learning framework. Further details outlining the specifics of each experiment’s environment and setup are provided in the subsequent subsections.

#### 3.2.1. Experimental Environment and Setup for Pig Detection and Tracking

Three experiments were conducted to evaluate the effectiveness of the method in detecting and tracking multiple pigs within the pigpen. In the first experiment, the ByteTrack method, consisting of the YOLOX-x detector and BYTE algorithm tracker, was used to demonstrate its effectiveness in detecting and tracking multiple pigs in the pigpen. The model was trained for 200 epochs using stochastic gradient descent (SGD) as the optimizer and the default hyperparameters with a batch size of 2 and a learning rate set to ${1\times 10}^{-4}$. To compare the performance, two other experiments were carried out, substituting the YOLOX-x detector in the ByteTrack method with other recent detectors, namely the YOLOv8-l [108] and YOLO-NAS-l [109] models. In the ByteTrack experiment with the YOLOv8-l detector, the model was trained for 200 epochs using the SGD optimizer and default hyperparameters. On the other hand, the ByteTrack model employing the YOLO-NAS-l detector was trained for 250 epochs using the Adam optimizer and default hyperparameters, with a batch size of 4 and a learning rate of ${4\times 10}^{-4}$. For all three experiments, during the training process, the model weights were initialized from the COCO pretrained model, and their performance was evaluated using the standard MOT metrics defined in [110,111]. Table 3 summarizes the description of each evaluation metric, where ↑ denotes higher scores (indicating better performance) and ↓ denotes lower scores (indicate better performance).

#### 3.2.2. Experimental Environment and Setup for Pig Behavior Recognition

For pig behavior recognition, an ablation study was first performed to quantitatively evaluate the effects of the image sequence length on the proposed MnasNet0.5–LSTM model and to select the appropriate image sequence length based on the findings. Subsequently, additional experiments were conducted to compare the performance. In these experiments, recent efficient CNN models were used, namely lightweight models such as RepGhostNet [112], EfficientNetv2s [113], MobileNetV3Small [114], and MnasNet1.0. Each of these models was used as an appearance feature extractor in its corresponding CNN–LSTM variant, resulting in the RepGhostNet0.5–LSTM, EfficientNetv2s–LSTM, MobileNetV3Small–LSTM, and MnasNet1.0–LSTM models, respectively. In addition, other experiments were conducted using VGG16–LSTM [80] and ResNet50–LSTM [81] models, which were previously used for pig behavior recognition. It shall be noted that all of the models used the same input size (224 × 224 pixels) in the CNN and a single layer LSTM with 64 hidden units. For the training process, all of the models used a cross-entropy loss function and an SGD optimizer with a gamma value of 0.1 and an epsilon of 1 × 10^−9^, maintaining a learning rate of 0.0005, except for the MobileNetV3Small–LSTM model, where a learning rate of 0.001 was employed. To address class imbalance during training, a class-weights technique was employed within the loss function. Other specific hyperparameters for each model are presented in Table 4.

The performance of all models was compared based on their inference time and F1 score values [115], which were computed using the following equations:

(1) $$Precision=\frac{TP}{TP+FP}$$

(2) $$Recall=\frac{TP}{TP+FN}$$

(3) $$F1\;score=\frac{2\times Precision\times Recall}{Precision+Recall}$$

Here, true positive (*TP*) represents the true pigs’ behaviors accurately classified as true, false positive (*FP*) represents the false pigs’ behaviors inaccurately classified as true, and false negative (*FN*) represents the true pigs’ behaviors inaccurately classified as false.

#### 3.2.3. Experimental Environment and Setup for Pig Behavior Analysis

The Pycirclize library [116] was used to plot the chord diagram, while the Matplotlib library was used to generate the hexbin plot, pixel distance–time plot, and box plot. On the other hand, the Sequence-to-Pattern Generation (Seq2Pat) library [117], which offers a constraint-based sequential pattern mining algorithm [101], was used as a tool for mining sequential patterns from pigs’ behaviors. During the mining process, the support and time constraint thresholds were set at 0.4 and 0.3 s, respectively.

Furthermore, the FP growth algorithm [105] from the Sequential Pattern Mining Framework (SPMF) [118] was used to generate associative correlative patterns from pigs’ behaviors, using threshold values of 0.4, 0.85, and 1 for support, confidence, and lift, respectively.

### 3.3. Results

#### 3.3.1. Detection and Tracking Results

Table 5 shows the experimental results for pig detection and tracking using the ByteTrack method, which consists of the YOLOX-x detector and BYTE algorithm tracker. The results show that in terms of detection performance, the model achieved a recall and precision of 0.988 and 0.983, respectively, indicating the robust ability of the model in detecting multiple pigs in the pigpen. These findings are highly significant since they directly influence the capacity of the model to uniquely identify pigs.

Moreover, in terms of tracking performance, the model achieved a MOTA of 0.971, suggesting that the proposed model is capable of accurately tracking multiple pigs within the pigpen. Furthermore, the MT value was 134, demonstrating the effectiveness of the model in tracking the majority of pigs (~98.523%). Both the PT and ML values were equal to 1, signifying that only a few pigs were partially tracked, and a small number of pigs were mostly lost. Furthermore, the model attained a low IDS value of 18, showcasing its capability to continuously maintain the identification of most pigs across different frames. The good performance of the ByteTrack with YOLOX-x detector confirms that the method is efficient for detecting and tracking multiple pigs in a pigpen, which is an essential initial step in the system, as it plays a crucial role in subsequent behavior recognition and analysis.

The results of the other two experiments conducted for performance comparison are presented in Table 6. The ByteTrack model, comprising the YOLOX-x detector and BYTE algorithm tracker, was compared with two other models, substituting the YOLOX-x detector in the ByteTrack method with recent detectors (YOLOv8-l and YOLO-NAS-l). The experimental results show that the ByteTrack model with the YOLOX-x detector outperformed the other models across all evaluation metrics. These findings strongly suggest that the proposed model is significantly more accurate in detecting and uniquely identifying multiple pigs in the pigpen across the video frames compared with the ByteTrack model which replaces the YOLOX-x detector with the YOLOv8-l and YOLO-NAS-l detectors.

#### 3.3.2. Behavior Recognition Results

In this section, the results of two experiments are presented. The first experiment is an ablation study, which quantitatively evaluates the effect of varying the image sequence length on the proposed model, MnasNet0.5–LSTM, and selects the image sequence length that gives the best performance. The findings from the ablation study (Table 7) indicate that using an image sequence length of three yielded better results compared with other image sequence lengths. Therefore, a sequence length of three was selected for our proposed model.

Furthermore, the detailed results of the proposed model for pig behavior recognition in terms of recall, precision, and F1 score using the selected image sequence length are summarized in Table 8. The results show that the MnasNet0.5–LSTM model achieved a precision of 0.932, along with a recall and F1 score of 0.931, proving that it can accurately recognize pigs’ behaviors. However, a lower accuracy was observed for behaviors such as “head-knocking-the-body”, “body-being-knocked-by-head”, and “head-to-head-knocking”, which can be attributed to the challenges associated with distinguishing these behaviors from others that exhibit similar postures, such as the “moving” behavior, potentially leading to misclassification.

The results of the second experiment, conducted for performance comparison, are tabulated in Table 9. The proposed model, MnasNet0.5–LSTM, was compared with other recent models (RepGhostNet0.5–LSTM, EfficientNetv2s–LSTM, MobileNetV3Small–LSTM, and MnasNet1.0–LSTM) as well as with the models previously used for pig behavior recognition (VGG16–LSTM and ResNet50–LSTM). The experimental results show that the MnasNet0.5–LSTM model outperformed all of the other models. The MnasNet0.5–LSTM model only showed a slight drop of 0.004 in the F1 score when compared with the MnasNet1.0–LSTM model. However, as detailed in Table 10, it is evident that the proposed model significantly reduced the number of parameters to 2,922,613 and decreased the execution time to 1.534 s per sequence, making it smaller and faster than the MnasNet1.0–LSTM model. Despite the considerable reduction in the number of parameters, the proposed model achieved good performance with minimal inference time, which can ensure precise recognition of different pigs’ behaviors while also enabling fast inference.

#### 3.3.3. Pig Behavior Analysis Results

This section presents the results of both descriptive statistical analysis and data mining. These findings are divided into six main subsections, each of which is described below.

(1) Results of Aggressive Pigs’ Relationships Analysis

**Figure 2:**
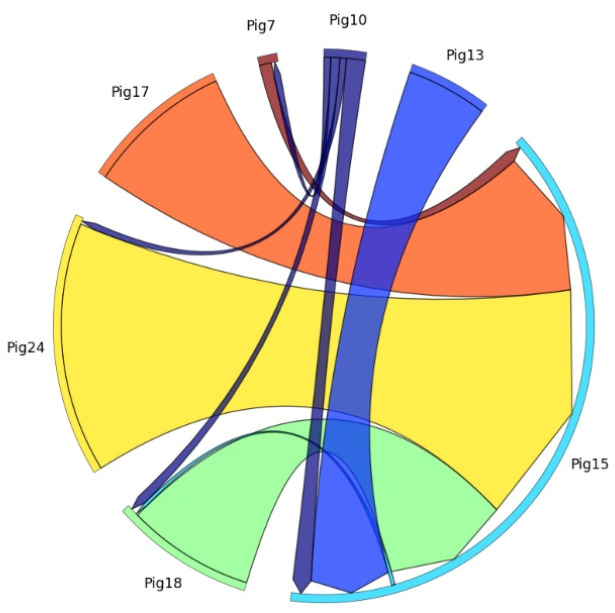
Chord diagram which enables visualization and identification of the interrelationships among aggressive pigs.

Figure 2 presents a chord diagram illustrating the aggressive interrelationships among pigs in the pigpen over a one-hour period when the pigs were active. Each aggressive pig is represented by an external node, and the relationships between them are depicted by arcs. The direction in which the arcs point indicates the victim pig, while the origin of the arrow represents the attacker in that relationship. The size of the arcs reflects the frequency of repeated attacks. As shown in Figure 2, Pig15 was targeted by all of the other pigs, making it the primary victim in the pigpen. Pig24 was the most frequent aggressor, followed by Pig17, Pig18, Pig13, Pig10, and Pig7. This observation suggests that Pig15 might be in a compromised state, potentially due to sickness, injury, or physical vulnerability, which makes it more susceptible to attacks from its peers [119]. Even though Pig15 exhibited aggression by attacking only Pig18, the extent of the attack was relatively mild compared with other instances of aggression. This implies that Pig15 may be physically weaker and less aggressive than other pigs displaying aggressive behavior.

These findings are significant as they can assist pig farmers in identifying both attacker and victim pigs, understanding their aggressive relationships, and assessing the degree of aggression. This information can ultimately guide pig farmers in taking appropriate action such as separating the aggressive pigs or improving pigpen conditions to address the aggressive behaviors in the pigpen.

(2) Results of Aggressive Behaviors Locations Analysis

**Figure 3:**
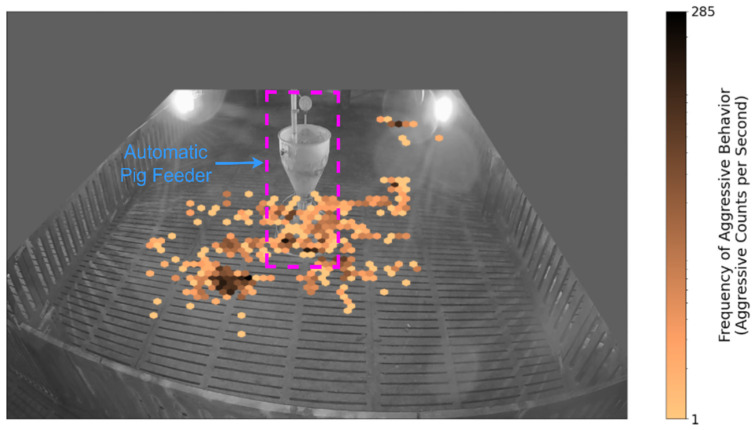
Hexbin plot which enables visualization of the locations where aggressive behaviors occurred in the pigpen.

The scenario depicted in Figure 3 represents the locations where aggressive behaviors occurred within the pigpen over a one-hour duration. As shown in the figure, the majority of aggressive incidents among pigs took place around or near the feeding area, with only a few occurrences observed far from it. These findings suggest that the primary cause of aggression may be related to food shortage, dominance battles over access to food, and competition for limited feeding space [11,120]. Understanding these underlying causes can guide pig farmers in devising strategies to mitigate aggression, such as adjusting feeding schedules or creating a more structured feeding environment to alleviate competition among pigs.

(3) Results of Pigs’ Movements Analysis

**Figure 4:**
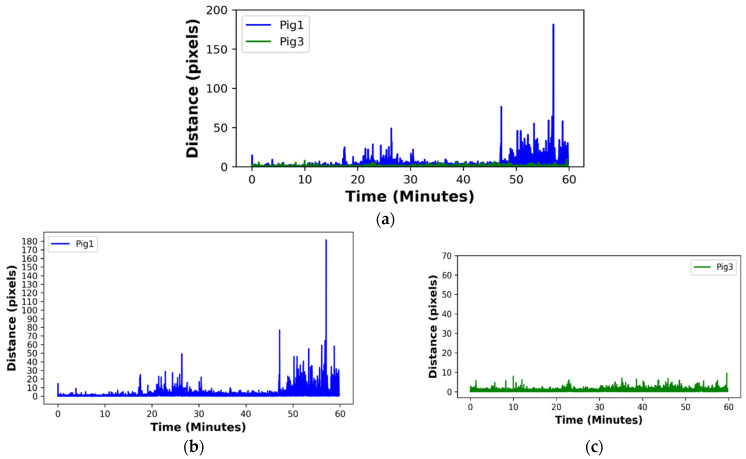
Pixel distance–time plot which enables visualization of the pigs’ movement patterns: (**a**) combined pixel distance–time plot for Pig1 and Pig3, (**b**) pixel distance–time plot for Pig1, and (**c**) pixel distance–time plot for Pig3.

The pixel distance–time plot is presented in Figure 4, where Figure 4a shows the combined plot for Pig1 and Pig3, enabling easy comparison of pixel movements among the pigs. Meanwhile, Figure 4b,c provide a separate plot for each pig, allowing clear visualization of their individual movements. As shown in Figure 4b, Pig1 exhibited a higher level of activity, reaching a peak distance above 175.0 pixels around the 55th to 60th minute, signifying more substantial movement compared with Pig3. Moreover, most of the time, Pig1 displayed higher activity levels than Pig3, denoting a greater overall activity. These observations imply that Pig1 is more likely to be a healthy pig [121]. By contrast, as presented in Figure 4c, Pig3 showed reluctance in movement or maintained a low level of locomotion, with the majority of its covered distance staying below 25 pixels. This suggests that Pig3 may be more likely to have welfare issues such as illness, leg weakness, and lameness [121,122].

These behavioral patterns are important as they can assist pig farmers in understanding the activity levels of pigs, identifying restlessness, and recognizing reduced movement, all of which may indicate the pigs’ health and welfare status [11].

(4) Results of Aggressive Behavior Variations Analysis

**Figure 5:**
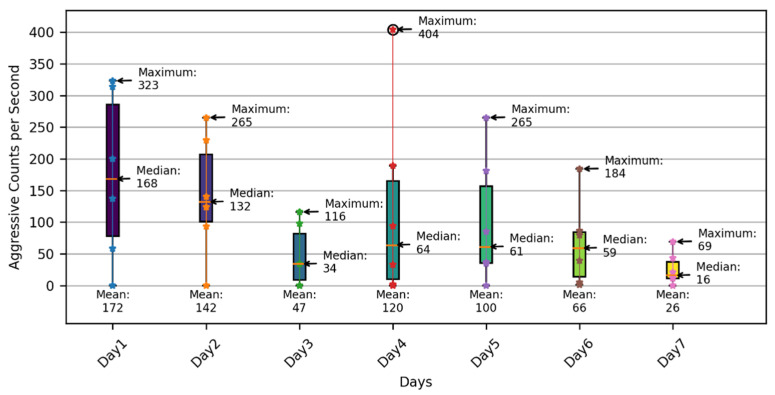
Box plot illustrating the variations of the pigs’ aggressive behaviors.

Figure 5 shows a box plot that highlights the variations in pigs’ aggressive behaviors throughout a week, based on data captured at different time intervals (08:00 to 10:00, 12:00 to 14:00, and 16:00 to 18:00), covering the periods when the pigs were active [123]. The results show that the aggressive behaviors decreased during the first three days, where the median and mean values decreased from 168 and 172 aggressive counts per second to 34 and 47 aggressive counts per second, respectively. However, on the fourth day, there was a slight increase in aggressive behavior compared with the third day, with a potential outlier of 404 aggressive counts per second, indicating unusual aggressiveness that might necessitate further investigation to identify the causes. Moreover, the results demonstrate that aggressive behaviors further decreased during the last three days, where the median and mean values decreased from 61 and 100 aggressive counts per second to 16 and 26 aggressive counts per second, respectively. In fact, as depicted in Figure 5, there was a noticeable decrease in the pigs’ aggressive behavior throughout the week, which may imply an improvement in the pigs’ living conditions, including adequate space and resources, the establishment of social hierarchy, and environmental enrichment [16,53,87]. Therefore, these findings enable pig farmers to comprehend the variations in pigs’ aggressive behaviors over time, detect unusual spikes in aggressiveness, implement necessary measures to address the identified issues, and ultimately maintain the pigs’ living conditions.

(5) Results of Behavioral Sequential Pattern Mining

Table 11 presents the results of mining sequential behavioral patterns in pigs for the data captured during daytime when the pigs were active over a three-week period. A total of 15 behavioral sequential patterns were found, varying in sequence size from 2 to 3. The results show that patterns 1 and 2 stood out with a support value of 0.586, signifying the most prevalent behavioral sequential patterns. These patterns predominantly involved transitions between “sleeping” and “sitting-resting” behaviors in sequence, or vice versa, representing normal behavior in healthy pigs [124]. In addition, patterns 6, 12, and 13 demonstrated that “eating” behavior was followed by aggressive behavior in a sequential manner, with a maximum support value of 0.462. This emphasizes that many pigs exhibit aggressive behavior after they start feeding, potentially indicative of problems such as insufficient food and feeding space in the pigpen [125,126], as well as inadequate nutrient supply in the food provided [127]. These findings further confirm the effectiveness of using both descriptive statistical analysis and data mining techniques. In fact, while the hexbin plot in (2) of Section 3.3.3 reveals the area where aggression mostly occurs, behavioral sequential patterns provide more detailed evidence about the behavior leading to aggression. Lastly, patterns 11 and 15 show that “eating” behavior was followed by “sleeping” behavior in sequence, with a maximum support value of 0.432. This highlights that many pigs tend to sleep after eating, which may be attributed to the digestive process, potentially signifying normal behavior in healthy pigs [128]. Identifying these patterns can be helpful as it enables pig farmers to gauge and understand normal exhibited behaviors as well as the causes of aggressive behavior, such as inadequate nutrient supply in the food provided and the lack of food and feeding space in the pigpen. All of these factors can assist pig farmers in developing solutions aimed at addressing observed deviations or abnormalities, enhancing the feeding environment and food quality, and maintaining the pigs’ living conditions. This can ultimately help reduce aggressive behaviors among pigs, improving their health and welfare [54,55].

(6) Results of Association Rule Mining on Pigs’ Behaviors

Table 12 shows the results of association rule mining on pigs’ behaviors for the data captured during the same period as in (5) above. A total of 15 patterns were generated, with an antecedent size ranging from 1 to 3 and a consequent size of 1. Moreover, all generated patterns had a confidence value above 0.850, implying a high degree of reliability for pig farmers to have a considerable level of trust. In addition, the lift value (greater than 1) signifies a notably stronger co-occurrence of these behaviors than would typically be anticipated, helping pig farmers in observing and understanding these coinciding behaviors for better management of such occurrences.

The results reveal that pattern 2 was the most associative and positively correlated behavioral pattern, with a support value of 0.637, indicating that many pigs spent their time in “resting” behavior. Furthermore, pattern 4 showed that “body-being-knocked-by-head” was associated and positively correlated with “sitting-resting” behavior, with a support value of 0.412. This pattern highlights that, when a pig becomes victim of an attack, the pig is more likely to rest due to injuries, lesions, and pain [103]. This signifies a severe problem for many pigs that might necessitate intervention due to its support value. Providing such behavioral patterns can help farmers gauge the effects of an aggression, prompting essential actions to address the issue such as enhancing pen layouts, reducing overcrowding, and mitigating any aggression sources among pigs. In addition, patterns 2, 3, 5, 6, 10, and 11 show the associations and positive correlations among normal pig behaviors. Understanding these behavioral patterns can significantly assist pig farmers in enhancing and maintaining informed decision-making regarding nutrition, housing, and health and welfare management. For instance, in pattern 5, “eating” and “moving” behaviors were associated and positively correlated with “sleeping” behavior (with a support value of 0.413), signifying that an active pig tends to rest upon receiving food. This finding indicates good welfare among pigs [61], enabling pig farmers to maintain the current farm layout, housing, and food, thereby enhancing the optimal living standards and productivity of the pigs. Therefore, providing farmers with information about pigs’ associative and correlative behavioral patterns is of paramount importance. This information can help pig farmers comprehend and address issues to improve farming practices, which will ultimately enhance the welfare and productivity of their pigs.

#### 3.3.4. System Graphical User Interface

**Figure 6:**
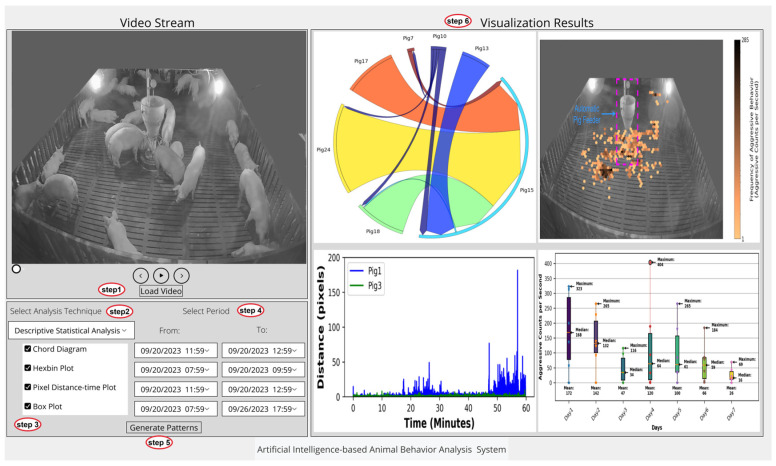
Descriptive statistical analysis interface: proposed system screenshot.

**Figure 7:**
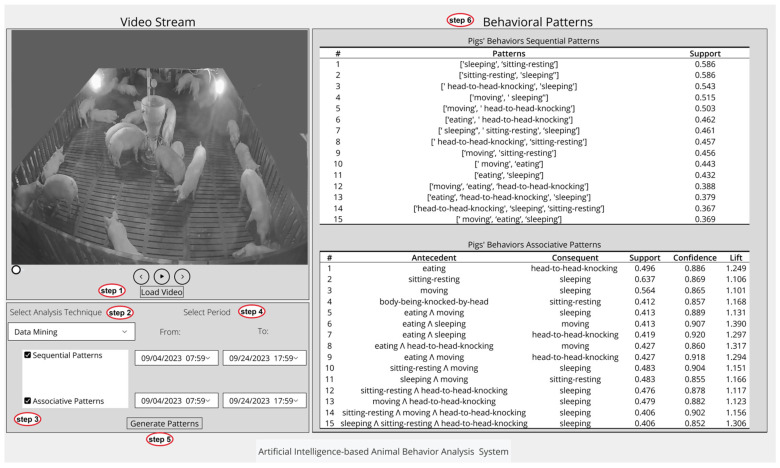
Data mining interface: proposed system screenshot.

A graphical user interface (GUI) was developed to assist pig farmers in conveniently accessing information that can effectively help them understand and manage their pigs’ health and welfare. This GUI enables them to visualize patterns related to aggressive pigs’ relationships, their locations and variations, as well as pigs’ movements using descriptive statistical analysis, as depicted in Figure 6. In addition, the GUI is an interactive tool for pig farmers to visualize sequential and associative behavioral patterns through data mining techniques, as shown in Figure 7.

To utilize this feature, users first load the video stream and select their desired analysis technique: either descriptive statistical analysis or data mining. This process is illustrated in figures as step 1 and step 2, respectively. Upon selection, corresponding patterns to be generated for the chosen technique are displayed, allowing users to select one or more by checking the respective boxes; this is depicted as step 3 in figures. Subsequently, in step 4, users must specify the intended time frame for each selected pattern. In step 5, users generate the patterns by clicking on the “Generate Patterns” button. These patterns are then displayed as visualization results or behavioral patterns, depending on the selected analysis technique, as shown in step 6.

## 4. Discussion

With the development of PLF, several studies have utilized video sensors coupled with deep learning to recognize pigs’ behavior. While these studies have yielded promising results, they often fall short in providing farmers with easily interpretable and actionable insights for effective management of their livestock. This limitation arises as it requires a significant amount of time and effort for farmers to read through behavior logs manually, often resulting in the oversight of crucial information relevant to their pigs’ health and welfare. In addressing this challenge, this paper proposes an approach that utilizes the YOLOX model and BYTE algorithm for detection and tracking of individual pigs, alongside the MnasNet-LSTM model for recognizing pigs’ behavior. Additionally, the method integrates descriptive statistical analysis and data mining techniques to provide farmers with visualized information and behavioral patterns. Consequently, it offers a viable solution for farmers, facilitating a comprehensive understanding and effective management of their pigs’ health and welfare.

By providing farmers with readily accessible and interpretable insights into their pigs’ behavior, it facilitates informed decision-making and proactive intervention to maintain and manage animal health and welfare. Furthermore, the adoption of such technology stands to enhance overall farm productivity and sustainability by optimizing resource allocation and minimizing the risk of aggressive behaviors, disease outbreaks, or suboptimal management practices.

## 5. Conclusions

Continuous monitoring of pigs’ behaviors can assist in identifying subtle changes that precede or accompany subclinical and clinical signs of diseases or injuries, thereby helping pig farmers in understanding and managing the health and welfare of their pigs. To equip farmers with appropriate solutions, we proposed a system that can automatically monitor, identify, and track individual pigs, recognize their behaviors, and analyze the tracking and behavior information to provide visualized information and behavioral patterns. This can effectively assist pig farmers in understanding and managing the health and welfare of their pigs. The system consists of four modules: (1) data acquisition module, (2) pig detection and tracking module, (3) pig behavior recognition module, and (4) pig behavior analysis module. The first module continuously receives a video stream from an infrared camera installed at the top of the pigpen, which monitors multiple pigs, and then forwards the video stream to the second module. Upon receiving the video, the second module uses the YOLOX-x detector to detect pigs, followed by the BYTE track algorithm to uniquely identify them by assigning a tracking ID to the detected pigs and performing an association between them across video frames. Subsequently, the pigs’ bounding boxes and IDs are stored as tracking information in the logged data and are also used to identify ROIs representing each pig in the pigpen, which are then used to crop their images and forward them to the third module. In the third module, the MnasNet–LSTM model is applied to the received sequences of cropped pig images to recognize the behavior of each specific pig and save the recognized behaviors as log data for further analysis. Finally, in the fourth module, the tracking information and recognized behaviors of the pigs are analyzed using descriptive statistical analysis and data mining techniques to provide pig farmers with visualized information and pattern mining results that can help them effectively understand and manage the health and welfare of their pigs.

Based on the experimental results, in terms of detection performance, our system achieved a recall and precision of 0.988 and 0.983, respectively. In terms of tracking performance, our system achieved a MOTA of 0.971. Our system also attained an F1 score of 0.931 for behavior recognition. These findings reveal that the method can robustly and accurately detect and track multiple pigs in the pigpen, as well as recognize their behaviors. Furthermore, the experiments demonstrated the effectiveness of descriptive statistical analysis and data mining in providing visualized information and behavioral patterns that can effectively help pig farmers understand and manage the health and welfare of their pigs. In future research, we intend to focus on improving the pigs’ tracking and behavior recognition performance by introducing multiview cameras in order to enhance the visibility of pigs from various angles, directions, and positions. In addition, since some pigs’ vocalizations are associated with health and welfare issues such as postweaning multisystemic wasting syndrome (PMWS), porcine reproductive and respiratory syndrome (PRRS), mycoplasma hyopneumoniae (MH), and aggression; as such, we will also explore the use of sound data to introduce a multimodal system for monitoring pigs’ health and welfare. Finally, in addition to using standard metrics, we will explore the possibility of using additional statistical tests on the selected models to ensure a more rigorous validation of the selected technique.

## Figures and Tables

**Table 1:** Some of the recent studies related to pig behavior recognition published from 2019 to 2023.

Detection and Tracking	Behavior Recognition Method	Targeted Behaviors	Purpose	Analysis of Recognized Behaviors and Information Extraction	Analysis and Information Extraction Technique(s)	Ref.
None	Traditional machine learning	* Posture	To validate the proposed method for posture detection under commercial farm conditions.	No	Not applicable	[37]
Detection	Deep learning	Drinking, urination, and mounting behaviors	To provide a solution to monitor pigs’ behaviors in real time.	No	Not applicable	[38]
Detection and tracking	Deep learning	* Posture and drinking behavior	To monitor subtle changes in pigs’ posture and drinking behavior to manage the pigs’ health and welfare.	Yes	Descriptive statistical analysis	[39]
None	Deep learning	Aggressive behavior	To provide a low-cost solution for farmers to monitor aggressive behavior.	No	Not applicable	[40]
Detection	Deep learning	* Posture	To monitor and assess pigs’ behavioral response under different heat conditions.	No	Not applicable	[41]
Detection	Deep learning	* Posture	To improve the accuracy of recognizing pig posture.	No	Not applicable	[42]
Detection	Deep learning	* Posture	To improve the performance of posture recognition in order to monitor pigs within enclosures.	No	Not applicable	[43]
None	Deep learning	Feeding and drinking behaviors	To provide a low-cost solution for monitoring feeding and drinking behaviors.	No	Not applicable	[44]

* Posture refers to exhibited behaviors that include some or all of the following: standing, sitting, sternal lying, lateral lying, prone, and sidling.

**Table 2:** Pig behavior description and dataset counts used for experiments on behavior recognition models.

Category	Behavior	Description	Number of Instances (Sequences of Three Images)
Normal behaviors	eating-drinking	Positioned with the head in a feeder.	13,605
	sleeping	Lying with the head on the floor without being attacked by other pigs.	36,373
	sitting-resting	Partly erected on stretched front legs with back end of the body contacting the floor.	41,263
	moving	Upright body position on extended legs with hooves only in contact with the floor.	8874
Aggressive behaviors	belly-nosing	Rubbing another pig’s belly with up-and-down snout movements.	3607
	being-belly-nosed	The belly of one pig is being rubbed with the snout of another pig.	3288
	tail-biting	Nibbling, sucking, or chewing the tail of another pig.	798
	being-tail-bitten	The tail of one pig is being nibbled, sucked, or chewed by another pig.	807
	ear-biting	Nibbling, sucking, or chewing the ear of another pig.	685
	being-ear-bitten	The ear of one pig is being nibbled, sucked, or chewed by another pig.	709
	head-knocking-the-body	Hitting another pig’s body with the head or snout.	4301
	body-being-knocked-by-head	The body of one pig is being hit with the head or snout of another pig.	3217
	head-being-knocked	Pigs hitting each other’s head or snout.	4807
	Total		122,334

**Table 3:** Metrics used to evaluate pig detection and tracking performance.

Metric	Description
Rcll (↑)	Recall
Prcn (↑)	Precision
GT	Ground truth of ID to be tracked
MT (↑)	Number of IDs mostly tracked
PT (↓)	Number of IDs partially tracked
ML (↓)	Number of IDs mostly lost
IDS (↓)	Number of ID switches
MOTA (↑)	Multi-object tracking accuracy

**Table 4:** Hyperparameters used for experiments using different models for pig behavior recognition.

Model	CNN Output Size	LSTM Input Size	Number of Epochs
VGG16–LSTM	4096	1×3×4096	40
ResNet50–LSTM	2048	1×3×2048	50
RepGhostNet0.5–LSTM	1280	1×3×1280	60
EfficientNetv2s–LSTM			40
MobileNetV3Small–LSTM			40
MnasNet1.0–LSTM			60
MnasNet0.5–LSTM			60

**Table 5:** Overall results for multiple pig detection and tracking using the proposed model (ByteTrack with YOLOX-x detector).

	Number of Frames	Rcll↑	Prcn↑	GT	MT↑	PT↓	ML↓	IDS↓	MOTA↑
Video00	1038	0.986	0.999	28	28	0	0	5	0.984
Video01	1324	0.992	1.000	25	24	1	0	2	0.991
Video02	1200	0.997	0.970	21	21	0	0	3	0.966
Vide03	1200	0.996	0.998	28	28	0	0	6	0.994
Video04	1600	0.962	0.939	18	17	0	1	2	0.900
Video05	1207	0.998	0.997	16	16	0	0	0	0.995
Overall	7569	0.988	0.983	136	134	1	1	18	0.971

**Table 6:** Comparison of the pig detection and tracking performance using the ByteTrack with different detectors.

Model	Number of Frames	Rcll↑	Prcn↑	GT	MT↑	PT↓	ML↓	IDS↓	MOTA↑
Bytetrack with YOLOv8-l	7569	0.888	0.961	136	104	23	9	139	0.851
ByteTrack with YOLO-NAS-l		0.964	0.978		125	8	3	75	0.941
ByteTrack with YOLOX-x		**0.988**	**0.983**		**134**	**1**	**1**	**18**	**0.971**

**Table 7:** F1 score results obtained from the ablation study for pig behavior recognition using the MnasNet0.5–LSTM model with various image sequence lengths.

Image Sequence Length	F1 Score
3	**0.931**
4	0.915
5	0.909
6	0.921
7	0.896
8	0.918
9	0.903
10	0.869

**Table 8:** Pig behavior recognition performance of the proposed model in terms of precision, recall, and F1 score.

Behavior	Precision	Recall	F1 Score	Data Count
eating-drinking	0.944	0.957	0.951	2255
sleeping	0.970	0.984	0.977	5034
sitting-resting	0.942	0.930	0.936	4863
moving	0.842	0.904	0.872	1871
belly-nosing	0.992	0.974	0.983	774
being-belly-nosed	0.997	0.974	0.986	774
tail-biting	0.963	0.959	0.961	242
being-tail-bitten	0.968	0.884	0.924	242
ear-biting	0.916	0.956	0.935	159
being-ear-bitten	0.974	0.949	0.962	158
head-knocking-the-body	0.756	0.802	0.778	756
body-being-knocked-by-head	0.892	0.683	0.774	723
head-to-head-knocking	0.876	0.876	0.876	1051
Weighted average	0.932	0.931	0.931	18,902

**Table 9:** Comparison of the pig behavior recognition performance for all models tested in this study using the F1 score.

Behavior	F1 Score						
	VGG16–LSTM	ResNet50– LSTM	RepGhost Net0.5–LSTM	Efficient Netv2s–LSTM	MobileNetV3 Small–LSTM	MnasNet 1.0–LSTM	MnasNet 0.5–LSTM
eating-drinking	0.943	0.930	0.917	0.933	0.898	**0.958**	0.951
sleeping	**0.982**	0.927	0.925	0.968	0.962	0.980	0.977
sitting-resting	0.934	0.893	0.878	0.922	0.917	**0.942**	0.936
moving	0.861	0.779	0.764	0.803	0.846	**0.873**	0.872
belly-nosing	0.951	0.925	0.931	0.857	0.963	0.972	**0.983**
being-belly-nosed	0.987	0.955	0.936	0.987	0.981	**0.988**	0.986
tail-biting	0.948	0.890	0.881	0.877	0.869	0.957	**0.961**
being-tail-bitten	**0.942**	0.825	0.893	0.915	0.878	0.933	0.924
ear-biting	**0.975**	0.855	0.841	0.936	0.933	0.951	0.935
being-ear-bitten	**0.968**	0.959	0.962	0.930	0.954	0.949	0.962
head-knocking-the-body	0.777	0.795	0.754	0.773	0.777	**0.830**	0.778
body-being-knocked-by-head	0.717	0.730	0.685	0.746	0.695	0.757	**0.774**
head-to-head-knocking	0.861	0.795	0.770	0.820	0.814	0.869	**0.876**
Weighted average	0.926	0.883	0.871	0.905	0.904	**0.935**	0.931

**Table 10:** Comparison of the pig behavior recognition models in terms of the number of parameters and execution time.

	VGG16–LSTM	ResNet50–LSTM	RepGhost Net0.5–LSTM	Efficient Netv2s–LSTM	MobileNetV3 Small–LSTM	MnasNet 1.0–LSTM	MnasNet 0.5–LSTM
Number of parameters	135,335,309	28,125,047	3,018,069	22,163,837	3,213,849	5,087,413	2,922,613
Execution time (s)-Per sequence	6.861	3.851	0.922	7.315	2.191	2.742	1.534

**Table 11:** Results of behavioral sequential pattern mining.

#	Behavioral Sequential Pattern	Support
1	[‘sleeping’, ‘sitting-resting’]	0.586
2	[‘sitting-resting‘, ‘sleeping’]	0.586
3	[‘head-to-head-knocking’, ‘sleeping’]	0.543
4	[‘moving’, ‘sleeping’]	0.515
5	[‘moving’, ‘head-to-head-knocking’]	0.503
6	[‘eating’, ‘head-to-head-knocking’]	0.462
7	[‘sleeping’, ‘sitting-resting’, ‘sleeping’]	0.461
8	[‘head-to-head-knocking’, ‘sitting-resting’]	0.457
9	[‘moving’, ‘sitting-resting’]	0.456
10	[‘moving’, ‘eating’]	0.443
11	[‘eating’, ‘sleeping’]	0.432
12	[‘moving’, ‘eating’, ‘head-to-head-knocking’]	0.388
13	[‘eating’, ‘head-to-head-knocking’, ‘sleeping’]	0.379
14	[‘head-to-head-knocking’, ‘sleeping’, ‘sitting-resting’]	0.367
15	[‘moving’, ‘eating’, ‘sleeping’]	0.369

**Table 12:** Results of association rule mining on pigs’ behaviors.

#	Antecedent	Consequent	Support	Confidence	Lift
1	eating	head-to-head-knocking	0.496	0.886	1.249
2	sitting-resting	sleeping	0.637	0.869	1.106
3	moving	sleeping	0.564	0.865	1.101
4	body-being-knocked-by-head	sitting-resting	0.412	0.857	1.168
5	eating Λ moving	sleeping	0.413	0.889	1.131
6	eating Λ sleeping	moving	0.413	0.907	1.390
7	eating Λ sleeping	head-to-head-knocking	0.419	0.920	1.297
8	eating Λ head-to-head-knocking	moving	0.427	0.860	1.317
9	eating Λ moving	head-to-head-knocking	0.427	0.918	1.294
10	sitting-resting Λ moving	sleeping	0.483	0.904	1.151
11	sleeping Λ moving	sitting-resting	0.483	0.855	1.166
12	sitting-resting Λ head-to-head-knocking	sleeping	0.476	0.878	1.117
13	moving Λ head-to-head-knocking	sleeping	0.479	0.882	1.123
14	sitting-resting Λ moving Λ head-to-head-knocking	sleeping	0.406	0.908	1.156
15	sleeping Λ sitting-resting Λ head-to-head-knocking	moving	0.406	0.852	1.306

Λ represents a conjunction (logical AND) between behaviors in a generated pattern.

## Data Availability

The datasets presented in this article are not readily available as they are part of ongoing research.

## References

[1] Choe J.-H., Yang H.-S., Lee S.-H., Go G.-W. (2015). Characteristics of pork belly consumption in South Korea and their health implication. J. Anim. Sci. Technol..

[2] Korea Meat Trade Association (KMTA). http://www.kmta.or.kr/kr/data/stats_price_year.php.

[3] OECD (2021). Meat Consumption (Indicator). https://www.oecd-ilibrary.org/content/data/fa290fd0-en.

[4] Oh S.H., Whitley N.C. (2011). Pork production in China, Japan and South Korea. Asian-Australas. J. Anim. Sci..

[5] Statistics Korea. https://kostat.go.kr/anse/.

[6] Korea Rural Economic Institute (KREI) (2015). Agriculture in Korea.

[7] The World Bank Group Employment in Agriculture (% of Total Employment) (Modeled ILO Estimate)—Korea, Rep. https://data.worldbank.org/indicator/SL.AGR.EMPL.ZS?end=2021&locations=KR&start=1991&view=chart.

[8] Kim B.-R., Jun I., Yoon J.-Y., Min J.-H., Park M., Kim M.-J., Kim B., Kim J., Han J. (2010). The Current Situation of Korean Agriculture Employment and Future Labor Policy in Korean Agriculture.

[9] ADAP (1996). Swine Management Manual: Agricutural Instructional Materials.

[10] Holinger M., Früh B., Prunier A., Edwards S., Illmann G., Melišová M., Leeb C., Rudolph G. (2017). Improving Health and Welfare of pigs, A Handbook for Organic Pig Farmers.

[11] Zimmerman J.J., Karriker L.A., Ramirez A., Schwartz K.J., Stevenson G.W., Zhang J. (2019). Diseases of Swine.

[12] Díaz J.A.C., Diana A., Boyle L.A., Leonard F.C., McElroy M., McGettrick S., Moriarty J., Manzanilla E.G. (2017). Delaying pigs from the normal production flow is associated with health problems and poorer performance. Porc. Health Manag..

[13] Van Staaveren N., Calderón Díaz J.A., Garcia Manzanilla E., Hanlon A., Boyle L.A. (2018). Prevalence of welfare outcomes in the weaner and finisher stages of the production cycle on 31 Irish pig farms. Ir. Vet. J..

[14] Marchant-Forde J., Marchant-Forde R.M. (2005). Minimizing inter-pig aggression during mixing. Pig News Inf..

[15] Rivera-Benítez J.F., De la Luz-Armendáriz J., Gómez-Núñez L., Vargas F.D., Escatell G.S., Ramírez-Medina E., Velázquez-Salinas L., Ramírez-Mendoza H., Ayala M.A.C., Tufiño-Loza C. (2021). Swine health: History, challenges and prospects. Rev. Mex. Ciencias Pecu..

[16] Llonch P., Mainau E., Temple D., Manteca X. Aggression in Pigs and Its Welfare Consequences. https://awecadvisors.org/en/aggression-in-pigs-and-its-consequences-on-welfare/.

[17] Chen C., Zhu W., Norton T. (2021). Behaviour recognition of pigs and cattle: Journey from computer vision to deep learning. Comput. Electron. Agric..

[18] Racewicz P., Ludwiczak A., Skrzypczak E., Składanowska-Baryza J., Biesiada H., Nowak T., Nowaczewski S., Zaborowicz M., Stanisz M., Ślósarz P. (2021). Welfare health and productivity in commercial pig herds. Animals.

[19] Matthews S.G., Miller A.L., Clapp J., Plötz T., Kyriazakis I. (2016). Early detection of health and welfare compromises through automated detection of behavioural changes in pigs. Vet. J..

[20] Wirth S., Gebhardt-Henrich S.G., Riemer S., Hattendorf J., Zinsstag J., Hediger K. (2020). The influence of human interaction on guinea pigs: Behavioral and thermographic changes during animal-assisted therapy. Physiol. Behav..

[21] Zhang L., Gray H., Ye X., Collins L., Allinson N. (2019). Automatic individual pig detection and tracking in pig farms. Sensors.

[22] Zhuang X., Bi M., Guo J., Wu S., Zhang T. (2018). Development of an early warning algorithm to detect sick broilers. Comput. Electron. Agric..

[23] Cao L., Xiao Z., Liao X., Yao Y., Wu K., Mu J., Li J., Pu H. (2021). Automated chicken counting in surveillance camera environments based on the point supervision algorithm: Lc-densefcn. Agriculture.

[24] Sa J., Choi Y., Lee H., Chung Y., Park D., Cho J. (2019). Fast pig detection with a top-view camera under various illumination conditions. Symmetry.

[25] Wu D., Wang Y., Han M., Song L., Shang Y., Zhang X., Song H. (2021). Using a CNN-LSTM for basic behaviors detection of a single dairy cow in a complex environment. Comput. Electron. Agric..

[26] Bo Z., Atif O., Lee J., Park D., Chung Y. (2022). GAN-Based Video Denoising with Attention Mechanism for Field-Applicable Pig Detection System. Sensors.

[27] Kim J., Suh Y., Lee J., Chae H., Ahn H., Chung Y., Park D. (2022). EmbeddedPigCount: Pig Counting with Video Object Detection and Tracking on an Embedded Board. Sensors.

[28] González-Baldizón Y., Pérez-Patricio M., Camas-Anzueto J.L., Rodríguez-Elías O.M., Escobar-Gómez E.N., Vazquez-Delgado H.D., Guzman-Rabasa J.A., Fragoso-Mandujano J.A. (2022). Lamb Behaviors Analysis Using a Predictive CNN Model and a Single Camera. Appl. Sci..

[29] Lee J., Jin L., Park D., Chung Y. (2016). Automatic recognition of aggressive behavior in pigs using a kinect depth sensor. Sensors.

[30] Lodkaew T., Pasupa K., Loo C.K. (2023). CowXNet: An automated cow estrus detection system. Expert Syst. Appl..

[31] Cuan K., Zhang T., Huang J., Fang C., Guan Y. (2020). Detection of avian influenza-infected chickens based on a chicken sound convolutional neural network. Comput. Electron. Agric..

[32] Berckmans D. (2014). Precision livestock farming technologies for welfare management in intensive livestock systems. OIE Rev. Sci. Tech..

[33] Larsen M.L.V., Wang M., Norton T. (2021). Information technologies for welfare monitoring in pigs and their relation to welfare quality^®^. Sustainability.

[34] Norton T., Chen C., Larsen M.L.V., Berckmans D. (2019). Review: Precision livestock farming: Building “digital representations” to bring the animals closer to the farmer. Animal.

[35] Berckmans D. (2017). General introduction to precision livestock farming. Anim. Front..

[36] Halachmi I., Guarino M. (2016). Editorial: Precision livestock farming: A “per animal” approach using advanced monitoring technologies. Animal.

[37] Nasirahmadi A., Sturm B., Olsson A.C., Jeppsson K.H., Müller S., Edwards S., Hensel O. (2019). Automatic scoring of lateral and sternal lying posture in grouped pigs using image processing and Support Vector Machine. Comput. Electron. Agric..

[38] Zhang Y., Cai J., Xiao D., Li Z., Xiong B. (2019). Real-time sow behavior detection based on deep learning. Comput. Electron. Agric..

[39] Alameer A., Kyriazakis I., Bacardit J. (2020). Automated recognition of postures and drinking behaviour for the detection of compromised health in pigs. Sci. Rep..

[40] Mluba H.S., Lee J., Atif O., Park D., Chung Y. Lightweight Video-based Approach for Monitoring Pigs’ Aggressive Behavior. Proceedings of the Annual Conference of KIPS (ACK) 2021.

[41] Bonneau M., Poullet N., Beramice D., Dantec L., Canario L., Gourdine J.-L. (2021). Behavior Comparison During Chronic Heat Stress in Large White and Creole Pigs Using Image-Analysis. Front. Anim. Sci..

[42] Ji H., Yu J., Lao F., Zhuang Y., Wen Y., Teng G. (2022). Automatic Position Detection and Posture Recognition of Grouped Pigs Based on Deep Learning. Agriculture.

[43] Huang L., Xu L., Wang Y., Peng Y., Zou Z., Huang P. (2022). Efficient Detection Method of Pig-Posture Behavior Based on Multiple Attention Mechanism. Comput. Intell. Neurosci..

[44] Zhuang Y., Zhou K., Zhou Z., Ji H., Teng G. (2023). Systems to Monitor the Individual Feeding and Drinking Behaviors of Growing Pigs Based on Machine Vision. Agriculture.

[45] Vranken E., Berckmans D. (2017). Precision livestock farming for pigs. Anim. Front..

[46] Cornou C., Kristensen A.R. (2013). Use of information from monitoring and decision support systems in pig production: Collection, applications and expected benefits. Livest. Sci..

[47] Piñeiro C., Morales J., Rodríguez M., Aparicio M., Manzanilla E.G., Koketsu Y. (2019). Big (pig) data and the internet of the swine things: A new paradigm in the industry. Anim. Front..

[48] Han J., Kamber M., Pei J. (2012). Data Mining Concepts and Techniques.

[49] Witten I.H., Frank E., Hall M.A., Pal C.J. (2011). Data Mining: Practical Machine Learning Tools and Techniques.

[50] Garcia Fontes S., Gonçalves Morato R., Stanzani S.L., Pizzigatti Corrêa P.L. (2021). Jaguar movement behavior: Using trajectories and association rule mining algorithms to unveil behavioral states and social interactions. PLoS ONE.

[51] Branco T., de Moura D.J., de Alencar Nääs I., da Silva Lima N.D., Klein D.R., de Oliveira S.R.M. (2021). The Sequential Behavior Pattern Analysis of Broiler Chickens Exposed to Heat Stress. AgriEngineering.

[52] Hoorweg F.A., Vermeer H.M., Pedersen L.J., Spoolder H.A.M. (2022). Review on Hunger Induced Behaviours: Aggression and Stereotypies.

[53] Rhim S.J., Son S.H., Hwang H.S., Lee J.K., Hong J.K. (2015). Effects of mixing on the aggressive behavior of commercially housed pigs. Asian-Australas J. Anim. Sci..

[54] O’Driscoll K., O’Gorman D.M., Taylor S., Boyle L.A. (2013). The influence of a magnesium-rich marine extract on behaviour, salivary cortisol levels and skin lesions in growing pigs. Animal.

[55] Houghton E. Management and Breeding Strategies to Reduce Aggression. https://www.thepigsite.com/articles/management-and-breeding-strategies-to-reduce-aggression.

[56] Pig Progress US Study to Focus on Enriching Pig Environment. https://www.pigprogress.net/pigs/us-study-to-focus-on-enriching-pig-environment/.

[57] EFSA Panel on Animal Health and Welfare (AHAW) (2014). Scientific Opinion concerning a Multifactorial approach on the use of animal and non-animal-based measures to assess the welfare of pigs. EFSA J..

[58] Godyń D., Nowicki J., Herbut P. (2019). Effects of environmental enrichment on pig welfare—A review. Animals.

[59] Mkwanazi M.V., Ncobela C.N., Kanengoni A.T., Chimonyo M. (2019). Effects of environmental enrichment on behaviour, physiology and performance of pigs—A review. Asian-Australas J. Anim. Sci..

[60] Van De Weerd H., Ison S. (2019). Providing effective environmental enrichment to pigs: How far have we come?. Animals.

[61] Ludwiczak A., Skrzypczak E., Składanowska-Baryza J., Stanisz M., Ślósarz P., Racewicz P. (2021). How housing conditions determine the welfare of pigs. Animals.

[62] Brendle J., Hoy S. (2011). Investigation of distances covered by fattening pigs measured with VideoMotionTracker^®^. Appl. Anim. Behav. Sci..

[63] Zhang Y., Sun P., Jiang Y., Yu D., Weng F., Yuan Z., Luo P., Liu W., Wang X. (2022). ByteTrack: Multi-object Tracking by Associating Every Detection Box. Proceedings of the Computer Vision—ECCV 2022: 17th European Conference.

[64] Tan M., Chen B., Pang R., Vasudevan V., Sandler M., Howard A., Le Q.V. (2019). Mnasnet: Platform-aware neural architecture search for mobile. Proceedings of the 2019 IEEE/CVF Conference on Computer Vision and Pattern Recognition (CVPR).

[65] Luo W., Xing J., Milan A., Zhang X., Liu W., Kim T.K. (2021). Multiple object tracking: A literature review. Artif. Intell..

[66] Bewley A., Ge Z., Ott L., Ramos F., Upcroft B. Simple online and realtime tracking. Proceedings of the IEEE International Conference on Image Processing (ICIP).

[67] Wojke N., Bewley A., Paulus D. Simple online and realtime tracking with a deep association metric. Proceedings of the IEEE International Conference on Image Processing (ICIP).

[68] Feichtenhofer C., Pinz A., Zisserman A. (2017). Detect to Track and Track to Detect. Proceedings of the IEEE International Conference on Computer Vision.

[69] Braso G., Leal-Taixe L. (2020). Learning a Neural Solver for Multiple Object Tracking. Proceedings of the IEEE Computer Society Conference on Computer Vision and Pattern Recognition.

[70] Zhang Y., Wang C., Wang X., Zeng W., Liu W. (2021). FairMOT: On the Fairness of Detection and Re-identification in Multiple Object Tracking. Int. J. Comput. Vis..

[71] Zhang Y., Yu C., Liu H., Chen X., Lei Y., Pang T., Zhang J. (2022). An Integrated Goat Head Detection and Automatic Counting Method Based on Deep Learning. Animals.

[72] Ge Z., Liu S., Wang F., Li Z., Sun J. (2021). YOLOX: Exceeding YOLO Series in 2021. arXiv.

[73] Yassine A., Mabrouk B., Facciolo G., Grompone Von Gioi R., Davy A. (2022). An assessment of Multi Object Tracking on low framerate conditions. HAL.

[74] Seidenschwarz J., Braso G., Serrano V., Elezi I., Leal-Taixe L. (2023). Simple Cues Lead to a Strong Multi-Object Tracker. Proceedings of the 2023 IEEE/CVF Conference on Computer Vision and Pattern Recognition (CVPR).

[75] Wei B., Yu A., Dong Z., He Z. (2023). Video SAR Target Detection and Tracking Method Based on Yolov5+Bytetrack. Proceedings of the 2023 8th International Conference on Signal and Image Processing (ICSIP).

[76] Nasirahmadi A., Hensel O., Edwards S.A., Sturm B. (2016). Automatic detection of mounting behaviours among pigs using image analysis. Comput. Electron. Agric..

[77] Zhu W., Guo Y., Jiao P., Ma C., Chen C. (2017). Recognition and drinking behaviour analysis of individual pigs based on machine vision. Livest. Sci..

[78] Chen C., Zhu W., Liu D., Steibel J., Siegford J., Wurtz K., Han J., Norton T. (2019). Detection of aggressive behaviours in pigs using a RealSence depth sensor. Comput. Electron. Agric..

[79] Atif O., Lee J., Park D., Chung Y. (2023). Behavior-Based Video Summarization System for Dog Health and Welfare Monitoring. Sensors.

[80] Chen C., Zhu W., Steibel J., Siegford J., Wurtz K., Han J., Norton T. (2020). Recognition of aggressive episodes of pigs based on convolutional neural network and long short-term memory. Comput. Electron. Agric..

[81] Chen C., Zhu W., Steibel J., Siegford J., Han J., Norton T. (2020). Classification of drinking and drinker-playing in pigs by a video-based deep learning method. Biosyst. Eng..

[82] Peluso V., Rizzo R.G., Calimera A. (2020). Efficacy of topology scaling for temperature and latency constrained embedded convnets. J. Low Power Electron. Appl..

[83] Turner S.P., Farnworth M.J., White I.M.S., Brotherstone S., Mendl M., Knap P., Penny P., Lawrence A.B. (2006). The accumulation of skin lesions and their use as a predictor of individual aggressiveness in pigs. Appl. Anim. Behav. Sci..

[84] Haigh A., O’Driscoll K. (2019). Irish pig farmer’s perceptions and experiences of tail and ear biting. Porc. Health Manag..

[85] Zonderland J.J., Wolthuis-Fillerup M., van Reenen C.G., Bracke M.B.M., Kemp B., den Hartog L.A., Spoolder H.A.M. (2008). Prevention and treatment of tail biting in weaned piglets. Appl. Anim. Behav. Sci..

[86] Chou J.Y., O’Driscoll K., D’Eath R.B., Sandercock D.A., Camerlink I. (2019). Multi-step tail biting outbreak intervention protocols for pigs housed on slatted floors. Animals.

[87] Landsberg G.M., Denenberg S. Behavioral Problems of Swine—MSD Veterinary Manual. https://www.msdvetmanual.com/behavior/normal-social-behavior-and-behavioral-problems-of-domestic-animals/behavioral-problems-of-swine.

[88] Ala-Kurikka E., Heinonen M., Mustonen K., Peltoniemi O., Raekallio M., Vainio O., Valros A. (2017). Behavior changes associated with lameness in sows. Appl. Anim. Behav. Sci..

[89] Luo L., Reimert I., Middelkoop A., Kemp B., Bolhuis J.E. (2020). Effects of Early and Current Environmental Enrichment on Behavior and Growth in Pigs. Front. Vet. Sci..

[90] O’Malley C.I., Steibel J.P., Bates R.O., Ernst C.W., Siegford J.M. (2022). The Social Life of Pigs: Changes in Affiliative and Agonistic Behaviors following Mixing. Animals.

[91] Brown S.M., Peters R., Nevison I.M., Lawrence A.B. (2018). Playful pigs: Evidence of consistency and change in play depending on litter and developmental stage. Appl. Anim. Behav. Sci..

[92] O′Malley C.I., Steibel J.P., Bates R.O., Ernst C.W., Siegford J.M. (2021). Time budgets of group-housed pigs in relation to social aggression and production. J. Anim. Sci..

[93] Zaki M.J. (2001). SPADE: An efficient algorithm for mining frequent sequences. Mach. Learn..

[94] Srikant R., Agrawal R., Apers P., Bouzeghoub M., Gardarin G. (1996). Mining sequential patterns: Generalizations and performance improvements. Proceedings of the International Conference on Extending Database Technology.

[95] Huynh B., Trinh C., Huynh H., Van T.T., Vo B., Snasel V. (2018). An efficient approach for mining sequential patterns using multiple threads on very large databases. Eng. Appl. Artif. Intell..

[96] Gan W., Lin J.C.W., Fournier-Viger P., Chao H.C., Yu P.S. (2019). A survey of parallel sequential pattern mining. ACM Trans. Knowl. Discov. Data.

[97] Ayres J., Flannick J., Gehrke J., Yiu T. Sequential pattern mining using A bitmap representation. Proceedings of the Proceedings of the ACM SIGKDD International Conference on Knowledge Discovery and Data Mining.

[98] Pei J., Han J., Mortazavi-Asl B., Wang J., Pinto H., Chen Q., Dayal U., Hsu M.C. (2004). Mining sequential patterns by pattern-growth: The prefixspan approach. IEEE Trans. Knowl. Data Eng..

[99] Fournier-Viger P., Gomariz A., Campos M., Thomas R. Fast vertical mining of sequential patterns using co-occurrence information. Proceedings of the Pacific-Asia Conference on Knowledge Discovery and Data Mining.

[100] Xu Z., Lee J., Park D., Chung Y. (2017). Multidimensional analysis model for highly pathogenic avian influenza using data cube and data mining techniques. Biosyst. Eng..

[101] Hosseininasab A., van Hoeve W.J., Cire A.A. Constraint-based sequential pattern mining with decision diagrams. Proceedings of the 33rd AAAI Conference on Artificial Intelligence.

[102] Karabatak M., Ince M.C. (2009). An expert system for detection of breast cancer based on association rules and neural network. Expert Syst. Appl..

[103] Camerlink I. Why Avoid Aggression between Pigs?. https://www.pigprogress.net/health-nutrition/why-avoid-aggression-between-pigs/.

[104] RSPCA What Are the Animal Welfare Issues Associated with Pig Production. https://kb.rspca.org.au/knowledge-base/what-are-the-animal-welfare-issues-associated-with-pig-production/.

[105] Han J., Pei J., Yin Y., Mao R. (2004). Mining frequent patterns without candidate generation: A frequent-pattern tree approach. Data Min. Knowl. Discov..

[106] Hipp J., Güntzer U., Nakhaeizadeh G. (2000). Algorithms for association rule mining—A general survey and comparison. ACM SIGKDD Explor. Newsl..

[107] Biresaw T.A., Nawaz T., Ferryman J., Dell A.I. (2016). ViTBAT: Video tracking and behavior annotation tool. Proceedings of the 2016 13th IEEE International Conference on Advanced Video and Signal Based Surveillance (AVSS 2016).

[108] Ultralytics YOLOv8. https://github.com/ultralytics/ultralytics.

[109] Deci-AI SuperGradients YOLO-NAS. https://github.com/Deci-AI/super-gradients.

[110] Bernardin K., Stiefelhagen R. (2008). Evaluating multiple object tracking performance: The CLEAR MOT metrics. EURASIP J. Image Video Process..

[111] Ristani E., Solera F., Zou R., Cucchiara R., Tomasi C. (2016). Performance measures and a data set for multi-target, multi-camera tracking. Proceedings of the European Conference in Computer Vision (ECCV 2016).

[112] Chen C., Guo Z., Zeng H., Xiong P., Dong J. (2022). RepGhost: A Hardware-Efficient Ghost Module via Re-parameterization. arXiv.

[113] Tan M., Le Q.V. (2021). EfficientNetV2: Smaller Models and Faster Training. arXiv.

[114] Howard A., Sandler M., Chen B., Wang W., Chen L.C., Tan M., Chu G., Vasudevan V., Zhu Y., Pang R. Searching for mobileNetV3. Proceedings of the IEEE International Conference on Computer Vision.

[115] Powers D.M.W. (2011). Evaluation: From Precision, Recall and F-Factor to ROC, Informedness, Markedness and Correlation. J. Mach. Learn. Technol..

[116] Shimoyama Y. PyCirclize: Circular Visualization in Python. https://github.com/moshi4/pyCirclize.

[117] Wang X., Hosseininasab A., Colunga P., Kadıoğlu S., van Hoeve W.-J. (2022). Seq2Pat: Sequence-to-Pattern Generation for Constraint-Based Sequential Pattern Mining. Proc. AAAI Conf. Artif. Intell..

[118] Fournier-Viger P., Lin J.C.-W., Gomariz A., Gueniche T., Soltani A., Deng Z., Lam H.T., Berendt B., Bringmann B., Fromont É., Garriga G., Miettinen P., Tatti N., Tresp V. (2016). The SPMF Open-Source Data Mining Library Version 2. Proceedings of the European Conference on Machine Learning and Knowledge Discovery in Databases.

[119] Oldham L., Arnott G., Camerlink I., Doeschl-Wilson A., Farish M., Wemelsfelder F., Turner S.P. (2021). Once bitten, twice shy: Aggressive and defeated pigs begin agonistic encounters with more negative emotions. Appl. Anim. Behav. Sci..

[120] D’Alessio R.M., Hanlon A., O’Driscoll K. (2023). Comparison of single- and double-spaced feeders with regard to damaging behavior in pigs. Front. Vet. Sci..

[121] Li Y., Martin W., Heins B., Johnston L., Lazarus W., Tallaksen J. Early Detection of Sick Pigs in Organic Systems_UMN Extension. https://extension.umn.edu/small-scale-swine-production/early-detection-sick-pigs-organic-systems.

[122] The Pig Site, Recognising Disease on the Farm. https://www.thepigsite.com/disease-and-welfare/managing-disease/recognising-disease-on-the-farm#.

[123] Stäbler R., Patzkéwitsch D., Reese S., Erhard M., Hartmannsgruber S. (2022). Behavior of domestic pigs under near-natural forest conditions with ad libitum supplementary feeding. J. Vet. Behav..

[124] Nielsen S.S., Alvarez J., Bicout D.J., Calistri P., Canali E., Drewe J.A., Garin-Bastuji B., Gonzales Rojas J.L., Schmidt G., Herskin M. (2022). Welfare of pigs on farm. EFSA J..

[125] Vargas J.V., Craig J.V., Hines R.H. (1987). Effects of feeding systems on social and feeding behavior and performance of finishing pigs. J. Anim. Sci..

[126] Hansen L.L., Hagelsø A.M., Madsen A. (1982). Behavioural results and performance of bacon pigs fed “AD libitum” from one or several self-feeders. Appl. Anim. Ethol..

[127] Van Der Meer Y., Gerrits W.J.J., Jansman A.J.M., Kemp B., Bolhuis J.E. (2017). A link between damaging behaviour in pigs, sanitary conditions, and dietary protein and amino acid supply. PLoS ONE.

[128] Chen G., Qin W., Ding J., Wan M., Guo L., Wang W. (2017). Designing and validation of the remote monitoring system for pigs’ survival based on IOT technology. Sci. Agric. Sin..

